# Feasibility study of FDG PET as an indicator of early response to aromatase inhibitors and trastuzumab in a heterogeneous group of breast cancer patients

**DOI:** 10.1186/2191-219X-2-34

**Published:** 2012-06-25

**Authors:** Brenda F Kurland, Vijayakrishna K Gadi, Jennifer M Specht, Kimberly H Allison, Robert B Livingston, Eve T Rodler, Lanell M Peterson, Erin K Schubert, Xiaoyu Chai, David A Mankoff, Hannah M Linden

**Affiliations:** 1 Clinical Research Division, Fred Hutchinson Cancer Research Center, Seattle, WA, 98109, USA; 2 Department of Medicine, University of Washington, Seattle, WA, 98195, USA; 3 Department of Pathology, University of Washington, Seattle, WA, 98195, USA; 4 Section of Hematology-Oncology, University of Arizona Cancer Center, Tucson, AZ, 85719, USA; 5 Department of Radiology, University of Washington, Seattle, WA, 98195, USA

**Keywords:** FDG PET, Ki-67, Breast cancer, Aromatase inhibitor, Trastuzumab, Pharmacodynamic response, Early response

## Abstract

**Background:**

In breast cancer endocrine therapy, post-therapy Ki-67 assay of biopsy material predicts recurrence-free survival but is invasive and prone to sampling error. [^18^F]Fluorodeoxyglucose (FDG) positron emission tomography (PET) has shown an early agonist or ‘flare’ response to tamoxifen and estradiol, but has not been tested in response to estrogen-lowering aromatase inhibitors (AIs). We hypothesized that decreased agonistic response to AIs would result in early FDG uptake decline. We also measured early response to trastuzumab (T), another targeted agent for breast cancer with differing mechanisms of action. Our study was designed to test for an early decline in FDG uptake in response to AI or T and to examine association with Ki-67 measures of early response.

**Methods:**

Patients with any stage of newly diagnosed or recurrent breast cancer were eligible and enrolled prior to initiation (or resumption) of AI or T therapy. FDG PET and tissue biopsy were planned before and after 2 weeks of AI or T therapy, with pretreatment archival tissue permitted. Cutoffs of ≥20% decline in standardized uptake value (SUV) as FDG PET early response and ≤5% post-treatment expression as Ki-67 early response were defined prior to analysis.

**Results:**

Forty-two patients enrolled, and 40 (28 AI, 12 T) completed serial FDG-PET imaging. Twenty-two patients (17 AI, 5 T) had newly diagnosed disease, and 23 (14 AI, 9 T) had metastatic disease (5 newly diagnosed). Post-treatment biopsy was performed in 25 patients (63%) and was either refused or not feasible in 15. Post-treatment biopsy yielded tumor in only 17/25 cases (14 AI, 3 T). Eleven of 14 AI patients with post-therapy tissue showed FDG PET early response, and there was 100% concordance of PET and post-therapy Ki-67 early response. For the T group, 6/12 showed an FDG PET early response, including 2/3 patients with post-therapy biopsy, all with Ki-67 >5%.

**Conclusions:**

Substantial changes in FDG PET SUV occurred over 2 weeks of AI therapy and were associated with low post-therapy proliferation. SUV decline was seen in response to T, but few tissue samples were available to test association with Ki-67. Our results support further investigation of FDG PET as a biomarker for early response to AI therapy.

## Background

Breast cancer is a common, life-threatening malignancy [1]; its biology may be driven by estrogen receptor (ER) expression and/or by overexpression or amplification of the epidermal growth factor family receptor HER2. Endocrine therapies such as aromatase inhibitors (AI) and targeted monoclonal antibodies such as trastuzumab (T; Herceptin®, Genentech, South San Francisco, CA, USA) have dramatically improved breast cancer outcomes for patients with tumors bearing the appropriate marker for response: ER for AIs and HER2 for T. However, not all tumors bearing these receptors respond to targeted therapy. Furthermore, in the metastatic setting, initial response to targeted therapy is commonly followed by eventual disease progression and resistance to therapy. Additional predictive markers of treatment sensitivity and resistance could allow rapid identification of effective treatments and avoidance of futile therapy.

Ki-67, an immunohistochemical assay of tumor proliferation [2], has been demonstrated to be a biomarker of early response to chemotherapy [3] and endocrine therapy [4,5]. In a study of 158 patients undergoing endocrine therapy (anastrozole or tamoxifen or anastrozole + tamoxifen), higher Ki-67 after 2 weeks of therapy predicted shorter recurrence-free survival, with a hazard ratio of 1.95 (95% CI 1.23 to 3.07) for 2.7-fold higher Ki-67, controlling for the effects of tumor size and quantitative ER level [4]. Data regarding the predictive utility of Ki-67 and other markers for HER2-targeted therapy are more limited. *In vitro* studies have suggested that the HER2-targeting tyrosine kinase inhibitor lapatinib has direct effects on pathways involving proliferation [6], supporting Ki-67 as a candidate biomarker for early response to HER2-targeted therapy. However, other evidence suggests that the primary mechanism of the HER2-targeted monoclonal antibody trastuzumab is pro-apoptotic [6-8]. Further study is needed to understand the mechanisms of HER2-targeted therapy and to identify markers of early response [9].

Although the evidence is strong for Ki-67 as an early response biomarker for endocrine therapy, serial tissue biopsies are not always practical, and not all biopsies yield evaluable tumor tissue. Noninvasive imaging techniques which evaluate the entire tumor burden can overcome these obstacles and offer serial evaluation of tumor metabolic activity *in vivo*. 2-Deoxy-2-^18^F]fluoro-d-glucose positron emission tomography (FDG PET) holds promise as a marker of tumor glycolysis that may parallel Ki-67 [10,11] as an indirect measure of cell proliferation [12]. Serial FDG PET can serve as a pharmacodynamic marker of response to chemotherapy in several tumors, including breast [13-15]. FDG PET is widely available and has been shown to predict response to breast cancer-targeted therapy as well as chemotherapy. For example, an agonist response to endocrine therapy or introduction of estradiol results in a ‘metabolic flare’ of FDG uptake that predicts clinical response to endocrine therapy [16-18]. FDG PET may also show an early response to the HER2-targeted agent lapatinib [14]. Based upon these data, we hypothesized that reductions in estrogen levels induced by AI therapy would result in an early decline in FDG PET standardized uptake value (SUV). This pilot study also explored whether a similar response would be seen in HER2-targeted therapy with T.

To determine whether change in FDG PET SUV at 2 weeks into therapy concurs with the 2-week proliferative index (Ki-67), we paired imaging and tissue assays in two groups: patients with ER + disease initiating AI therapy and patients with HER2+ disease initiating T therapy. We hypothesized that change in FDG SUV would parallel post-therapy Ki-67.

## Methods

### Study design and patient population

Patients were screened and recruited from the breast oncology clinic at the University of Washington housed at the Seattle Cancer Care Alliance. Eligibility was broad, encompassing patients with *de novo* or recurrent disease planning therapy containing AI or T. No restrictions were placed on tumor stage, endocrine therapy history, or chemotherapy history. Washout periods for prior therapy were observed as clinically indicated. Inclusion criteria included biopsy-proven breast cancer with confirmation of ER or HER2 expression and lesions large enough (>3 cm diameter) to avoid FDG PET partial volume effects. Our primary endpoints were early response by FDG PET and tissue assay, rather than response by RECIST criteria [19], so patients with bone-dominant metastatic breast cancer were eligible if bone lesions were approximately 3 cm or larger and detected by FDG PET. Premenopausal patients could enroll in the AI arm if ovarian suppression was also administered, starting at least 2 weeks prior to AI. Patients with tumors which were both ER + and HER2+ were eligible for either arm, but single-agent therapy for the 2-week study period was required.

Patients underwent a pretreatment biopsy or consented to make a tissue block available from an earlier biopsy. A biopsy was performed after 2 weeks of therapy when feasible. FDG PET was performed pretreatment and after 2 weeks of therapy. After this 2-week brief therapeutic exposure (‘run-in’) test of single-agent therapy, patients then continued the single agent or added cytotoxic chemotherapy as planned. Neither study imaging nor 2-week study biopsy results were used to direct further treatment. Clinical response at 6 months was assessed using radiologic measurements and symptom report, but was not part of this analysis. Participants gave written informed consent, and the study was approved by the University of Washington Institutional Review Board.

### Tissue specimens and Ki-67

Baseline tissue was obtained as clinically indicated, from a diagnostic biopsy or remote biopsy of metastatic tissue. The 2-week study biopsy was taken from the breast when tumor was present, but permitted to be sampled from the skin, nodes, bone, or other sites as clinically indicated. Biopsy of multiple sites was not considered, out of concern for patient comfort. Nonconcurrent ‘baseline’ tumor blocks were not requested for patients who did not have follow-up biopsy results.

Paraffin-embedded blocks were analyzed at the University of Washington Pathology Laboratory for Ki-67 using a working dilution of 1:500 of MIB-1 antibody from DakoCytomation (Carpenteria, CA, USA). Although consensus guidelines had not been published at the time of analysis [2], Ki-67 assay followed the consensus protocol, with the exception of estimating the proliferation index from counts of 500 cells. Ki-67 was scored as the percentage of positively staining cells, based on counts of approximately 50 cells compared to standardized images and verified by a single observer blinded to the PET results. The Ki-67 assay is standard at our institution, and results were documented in the patients' medical record. However, the assays were performed in batches so post-therapy results were not routinely available to clinicians within a timeframe that could influence treatment. Status for other breast cancer markers (ER, PgR, HER2) was ascertained from the medical record, from the most recent assessment.

A post-therapy Ki-67 index of ≤5% was considered to confer a favorable prognosis. On a continuous scale for logged values, higher Ki-67 after 2 weeks of endocrine therapy has been found to be associated with shorter recurrence-free survival [4]. Cut points are desirable for eventual personalized therapy decisions, but are not yet standard due to differences in assay implementation and lack of data for cut point validation [2]. We chose a cut point of ≤5% as the lower limit of detection for our assay and a marker of cell cycle response [20].

### Imaging

FDG PET was performed on an ADVANCE PET scanner or a Discovery STE PET/CT scanner (GE Medical Systems, Waukesha, WI, USA), both in routine use at our center. Some patients' follow-up studies were performed on a different PET or PET/CT scanner, when the pre-therapy scanner was not available. However, scanners were regularly cross-calibrated using quantitative imaging phantom measurements. Patients underwent FDG PET or PET/CT using 7 to 10 mCi of FDG, infused over 1 min. Patient preparation instructions followed consensus guidelines [21], including fasting for 6+ h, maintaining hydration, and avoiding vigorous exercise for 24 h prior to scanning. Plasma glucose was measured prior to scanning, and the scan was not performed if glucose was >200 mg/dL. Imaging began 60 min after injection and consisted of five adjacent imaging fields covering neck to pelvis, with 7-min emission collections per field. Attenuation correction was performed from segmentation of transmission images (3 min per field, ADVANCE) or CT images (Discovery STE). Images were reconstructed using filtered backprojection with a smoothing filter, resulting in approximately 10-mm full width at half maximum resolution.

Images were analyzed prospectively by a single observer (DAM), with correlative anatomic imaging (typically CT) for assistance with tumor identification. If multiple lesions were present, a single index lesion for both FDG PET scans was chosen with the highest FDG uptake at baseline. When possible, a lesion amenable to post-therapy biopsy was chosen as the index lesion. The percent change in index lesion maximum FDG SUV compared to baseline was the primary analysis parameter for evaluating early response. An SUV decline of ≥20%, twice an accepted 10% test-retest reproducibility standard deviation [22], was considered to be attributable to treatment. This is a smaller threshold than the ≥30% and ≥0.8 unit decrease required for partial response under the PET Response Criteria in Solid Tumors (PERCIST) version 1.0 [23], but is justified by the short timeframe of treatment and the acceptance of lower thresholds in other consensus criteria [24].

### Statistical analysis

The nonparametric Wilcoxon rank sum test was used to compare distributions of AI and T groups for continuous measures such as SUV, Ki-67, and percent change in SUV. Spearman rank correlations were used to summarize associations between tissue and imaging measures. Other analyses of these pilot data were descriptive. The study was designed to test the ability of FDG PET to detect early response to AI or T (≥20% SUV decline) in 40% of patients, an approximate rate of clinical response to these targeted therapies. Assuming a sample size of 20 for each treatment group and 15 percentage point standard deviation for SUV decline, the power to observe FDG PET early response in at least 8/20 patients was 0.65 if the study population included 40% treatment responders. If 60% of the study population were responders to targeted therapy, the probability of observing FDG PET early response in 8+ of 20 patients was 0.97. Analyses were conducted using R version 2.13.1 (R Foundation for Statistical Computing, Vienna, Austria) and SAS/STAT software version 9.2 (SAS Institute, Inc., Cary, NC, USA).

## Results

### Patient characteristics

Forty-two patients were enrolled to the study, but one withdrew consent before the first PET scan and another, before the second PET scan. Thus, the analysis sample was 40 patients (28 undergoing an AI run-in, 12 undergoing T run-in). All 6 patients who enrolled with dual positive tumors (ER + and HER2+) opted for HER2-targeted therapy. One patient did not comply with AI therapy during the run-in period, leaving 39 early response-evaluable patients enrolled between December 2006 and October 2009. Table 1 describes patient characteristics for the AI and T cohorts. Seventeen AI-treated patients had *de novo* disease: 3 presented with metastatic disease, and 14 presented with stage I-III disease (nine of these patients were co-enrolled in a clinical trial of neoadjuvant therapy, NCT00194792, in which the AI run-in was followed by neoadjuvant chemotherapy + AI). The remaining 11 AI patients had recurrent stage IV disease up to 29 years after primary therapy. Six of these 11 AI patients had a history of endocrine therapy (tamoxifen, fulvestrant), including four with prior AI exposure. Five T-treated patients had *de novo* disease (two with metastatic disease at diagnosis). Five had recurrent disease newly diagnosed as stage IV, and two had recurrent metastatic disease. Four of the seven T-treated patients with recurrent disease had prior adjuvant T exposure, including one who had also received T for metastatic disease.

**Table 1:** **Patient characteristics, *n* ** **= 40**

	**Aromatase inhibitor ( *n* ** **= 28)**		**Trastuzumab ( *n* ** **= 12)**	
	** *n* (%)**	**Median** **(min, max)**	** *n* (%)**	**Median** **(min, max)**
Age, years	58 (35, 83)		48 (38, 71)	
Postmenopausal	21 (75%)		9 (75%)	
ER positive	28 (100%)		6 (50%)	
PgR positive	26 (93%)		6 (50%)	
HER2				
Negative	25 (89%)		0 (0%)	
Positive	0 (0%)		12 (100%)	
Equivocal/not done	3 (11%)		0 (0%)	
Disease status				
Newly diagnosed	17 (61%)		5 (42%)	
Recurrent	11(39%)		7 (58%)	
Disease stage				
Stage I-III (newly diagnosed)	14 (50%)		3 (25%)	
Metastatic (newly diagnosed)	3 (11%)		2 (17%)	
Metastatic (recurrent, newly stage IV)	5 (18%)		5 (41%)	
Metastatic (recurrent stage IV)	6 (21%)		2 (17%)	
Histology				
Ductal	14 (50%)		9 (75%)	
Lobular	9 (32%)		1 (8%)	
Ductal and lobular	2 (7%)		0 (0%)	
Unknown	3 (11%)		2 (17%)	
PET index lesion				
Breast	16 (57%)		5 (41%)	
Nodal/soft tissue	6 (21%)		3 (25%)	
Bone	1 (4%)		2 (17%)	
Visceral	5 (18%)		2 (17%)	

### FDG PET

**Figure 1:**
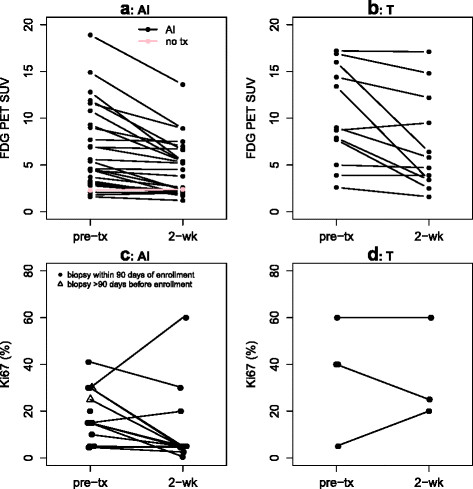
**Baseline and follow-up measures.** (**a**) AI therapy, FDG PET SUV. (**b**) T therapy, FDG PET SUV. (**c**) AI therapy, Ki-67% staining. (**d**) T therapy, Ki-67% staining.

Imaging findings are summarized in Table 2 and Figure 1. Baseline SUV had a similar range for AI (median 4.6, range 1.6 to 18.9) and T groups (median 8.9, range 2.6 to 17.2), but on average, values were somewhat lower for the AI group (*W* = 93, *p* = 0.03), as would be expected for ER-expressing tumors [25]. The median number of days between FDG PET scans was 18 days for the AI group and 15 days for the T group and ranged from 12 to 33 days. The maximum glucose level measured was 138 mg/dL, and for 39 patients with plasma glucose recorded at both scans, the median absolute difference between scans was 10 mg/dL (range 0 to 50 mg/dL). Time between FDG injection and scanning was available for 78 scans and was performed 55 to 65 min after injection for 58 (74%), with another 12 performed at about 45 min and 8 at 66 to 85 min after injection. Timing for the two scans differed by more than 20 min for three patients in the AI group, but in each case, scan timing would not have influenced early response classification (for example, one patient had a baseline SUV of 4.4 at 67.5 min and a post-therapy SUV of 3.8 at 45 min after injection. Any adjustment based on uptake time would attenuate the 14% decline toward 0%).

**Table 2:** **Imaging and Ki-67 results, *n* ** **= 40**

	**Aromatase inhibitor** **( *n* ** **= 28)**		**Trastuzumab** **( *n* ** **= 12)**	
	** *n* (%)**	**Median** **(min, max)**	** *n* (%)**	**Median** **(min, max)**
SUV_max_ of index lesion at baseline	28	4.6 (1.6, 18.9)	12	8.9 (2.6, 17.2)
Days between FDG PET scans	28	18 (12, 33)	12	15 (13, 29)
SUV_max_ of index lesion at follow-up	28	3.2 (1.2, 13.6)	12	5.3 (1.6, 17.1)
Percent change in SUV_max_	28	−28 (−60, 11)	12	−25 (−75, 9)
Patients with FDG PET early response (≥20% SUV decline)	20 (71%)		6 (50%)	
Ki-67 prior to run-in therapy (%)	22	15 (<5, 41)	5	40 (5, 60)
Days between successful biopsies	14	51 (16, 608)	3	29 (14, 49)
Ki-67 following run-in therapy (%)	14	5 (0.5, 60)	3	25 (20, 60)
Patients with Ki-67 ≤5% following run-in therapy	11 (79%)		0 (0%)	
Change in Ki-67	14	−8 (−26, 30)	3	0 (−15, 15)

**Figure 2:**
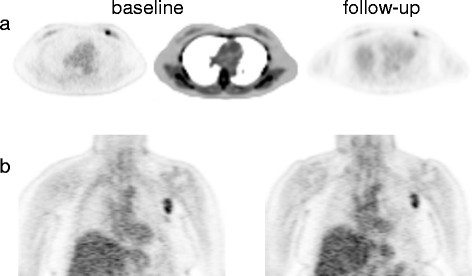
**Early FDG PET response examples**. (**a**) Trans-axial FDG PET views show left breast lesion with baseline SUV of 4.6 and follow-up SUV of 2.5 (46% decrease). Clinical response was observed after 4 months of neoadjuvant therapy + AI. (**b**) Coronal FDG PET views show left axillary lymph node with baseline SUV of 4.5 and follow-up SUV of 4.5 (0% change). This patient experienced progressive disease after 7 months of AI monotherapy.

FDG SUV declined in most patients on targeted therapy. Figure 1 shows a spaghetti plot of individual patients' FDG SUV for baseline and post-therapy for AI (Figure 1a) and T-treated (Figure 1b) groups undergoing approximately 2 weeks of targeted therapy. No patients showed increases in SUV greater than 11%, but 74% of AI patients (20/27) and 50% of T patients (6/12) had decreases in SUV of 20% to 75%. The noncompliant AI patient (pink line), who did not undertake any treatment between scans, had a baseline SUV of 2.3 and a follow-up SUV of 2.4. The distribution of percent change in SUV did not differ for the AI and T treatment groups (*W* = 158, *p* = 0.90). Examples of tumors with FDG SUV early response (≥20% decline) and non-response are shown in Figure 2.

### Ki-67

For all newly diagnosed patients (17 AI, 5 T), baseline biopsies were obtained within 3 months of the first FDG PET scan. For the remaining 11 AI patients, five baseline biopsies had been performed within 3 months, and the remaining 6 were archival. For the remaining seven T patients, five had undergone recent biopsies, and 2 samples were archival (5+ years old). Of these baseline biopsies, 13 (6 AI, 7 T) were not assessed for Ki-67 because the archival tissue could not readily be obtained, and follow-up Ki-67 was not acquired. Twenty-seven of 40 biopsies (68%) were of breast tissue. The remainder included the nodes (*n* = 3), liver (*n* = 3), and bone (*n* = 3). Baseline Ki-67 was >5% for 17/22 patients in the AI group (77%) and for 4/5 (80%) in the T group.

**Figure 3:**
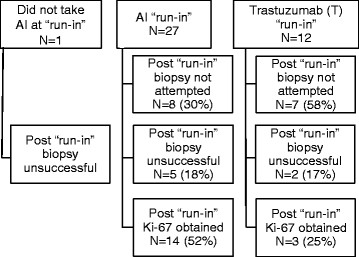
Flow chart for 40 study participants completing serial FDG PET.

Figure 3 demonstrates the compliance and yield of biopsy following a 2-week therapy run-in period. Biopsy was refused or not safely feasible for 15/40 patients (38%), all of whom had metastatic disease. Of the 25 biopsies performed, 8 (32%) failed to yield sufficient tumor tissue to assess Ki-67 (5 breast biopsies, 2 bone marrow/iliac crest, 1 axillary lymph node). The 17 successful 2-week biopsies (14 AI, 3 T) were all repeated samples from the baseline biopsy location, 16 from breast tissue and 1 from an axillary lymph node. Figure 1c (AI) and Figure 1d (T) show Ki-67 decline in the majority of patients on AI therapy. Following run-in, 11/14 of the AI group (79%) and 0/3 of the T group had K-67 ≤5%.

### Correlation of FDG PET and Ki-67 results

**Figure 4:**
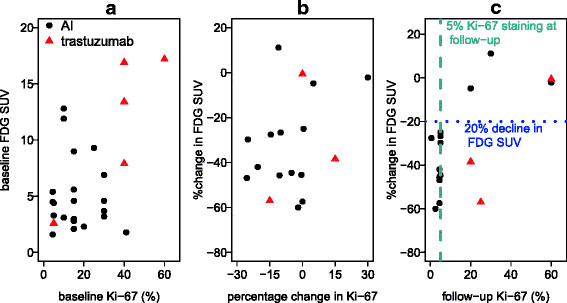
**Associations between imaging and tissue measures.** (**a**) Baseline FDG SUV and baseline Ki-67. (**b**) Percent change in FDG SUV and percentage change in Ki-67. (**c**) Percent change in FDG SUV and follow-up Ki-67.

We examined the correlation between change in SUV by FDG PET and tissue assay of Ki-67 by testing associations between baseline, 2-week absolute values, and percentage change. As shown in Figure 4a and discussed above, baseline SUV and Ki-67 were generally higher for most patients who were candidates for T therapy (red points) than for candidates for AI therapy. Otherwise, there was not a clear association between baseline levels for Ki-67 and FDG SUV (AI sample, Spearman rho = −0.07, *p* = 0.74). As shown in Figure 4b, change in Ki-67 and percent change in FDG SUV were also not strongly associated (AI sample, Spearman rho = 0.23, *p* = 0.44). However, the expected association between follow-up Ki-67 and percent change in FDG SUV was quite robust for the AI group (Figure 4c, AI sample Spearman rho = 0.77, *p* = 0.001). For all 11 patients in the AI group who showed an SUV decline of 20% or greater, the Ki-67 at follow-up was ≤5%; for all three AI patients who did not attain SUV reductions of 20% or greater, the Ki-67 at follow-up was >5%. The T group (red points) was too small to draw conclusions, but did not appear to show this pattern: two of three patients with serial biopsy had substantial reductions in FDG SUV (38% and 57%), but follow-up Ki-67 was >5% (20% and 25%).

## Discussion

FDG SUV demonstrated substantial decline over a 2-week course of AI therapy. This is consistent with the reasoning that if hormone-dependent breast cancers respond to estradiol challenge with an increase in FDG uptake [16-18], then tumors dependent upon the ER pathway for growth should display a decline in FDG uptake as AI therapy lowers estradiol levels. These early declines in FDG uptake corresponded to low post-therapy proliferation (Ki-67), which predicts early response to endocrine therapy [4]. For all 11 patients in the AI group who showed an SUV decline of 20% or greater, the Ki-67 at follow-up was ≤5%; for all three AI patients who did not attain SUV reductions of 20% or greater, the Ki-67 at follow-up was >5%. This supports the feasibility of serial FDG SUV as an early indicator of response to endocrine therapy.

For trastuzumab, 6 of 12 patients showed substantial decreases in FDG PET SUV, including 2 of 3 patients with serial biopsy, but none of the three 2-week biopsies had Ki-67 expression ≤5%. Although numbers are too small to draw conclusions, a less consistent pattern of change is perhaps not surprising. Earlier studies have suggested that T may mediate apoptotic pathways [7], rather than resulting in an early change in proliferation. In clinical practice and in most studies, T is given in combination with chemotherapy. Data emerging from recent neoadjuvant clinical trials suggest that HER2+ disease can be effectively targeted with additional agents aimed at HER2 such as lapatinib [26] and pertuzumab [27]. Further study is needed before drawing any conclusions about the efficacy of FDG PET as an early indicator of response to trastuzumab or other HER2-targeted therapies.

We did not see correspondence between change in FDG uptake and *change* in Ki-67 with AI therapy. Although post-therapy Ki-67 may be a better biomarker than the change in Ki-67 (in the context of predicting breast cancer recurrence) [4], other possible contributing factors are attributable to the preliminary nature of our pilot study. Of the 17 baseline tissue samples available for pre-post comparisons, 11 were archival (8 AI 3 T), with intervening therapies including cytotoxic chemotherapy, endocrine therapy, and HER2-targeted therapy. Small sample size and heterogeneity of disease characteristics and tissue biopsy sites may also confound our comparisons of imaging parameters to the change in Ki-67.

Another limitation of our study was the timing of imaging and biopsy. Our intentions were to assess the same lesion by imaging and biopsy and to schedule FDG PET scans before biopsy. However, clinical scheduling (diagnostic biopsy preceding PET scans) and feasibility concerns (i.e., imaging of lesions near a high-contrast organ or biopsy of an inaccessible tumor) had to be accommodated. We did not find evidence that post-biopsy inflammation strongly affected SUV. For three AI patients with *baseline* FDG PET within 21 days after biopsy of the index lesion (6, 13, 17 days), the percent changes in SUV were −28%, −7%, and −3%. For the three AI patients with *post-therapy* FDG PET within 21 days after biopsy (3, 5, 7 days), the percent changes in SUV were −14%, −47%, and −60%. If post-biopsy SUV were inflated, the opposite trend (greater decrease when the baseline scan occurred post-biopsy, lesser decrease when the post-therapy scan occurred post-biopsy) would be expected. Extension of run-in periods may be considered to avoid having post-therapy scans within a healing period after the baseline biopsy.

Our choice of a 20% threshold as attributable to therapy was motivated by our hypothesis that short-term exposure would produce a pharmacodynamic effect detectable by FDG PET and beyond the limits of agreement in test-retest studies. This is a different task than the estimation of clinical response, for which alternative measures such as PERCIST 1.0 have been suggested. Of note, 8 of the 20 AI group patients with early response by FDG PET SUV had decreases between 20% and 30%, and 4 of those had absolute change in SUV of less than 1.2. Alternative measures of FDG uptake may provide more robust evidence of a drug response [25]; however, such analysis was beyond the scope of this pilot study.

## Conclusions

This pilot study supports the use of FDG PET to examine early response to endocrine therapy and exposed challenges in collecting research biopsies in metastatic lesions. Early response patterns to AI therapy seen by imaging and biopsy were not apparent in response to T therapy, supporting that the findings for AI therapy were not due to technical issues in repeat imaging and/or biopsy. A further study in patients with *de novo* early stage disease, using paired serial imaging and tissue sampling to investigate early response to preoperative run-in targeted therapy, may determine whether imaging can replace tissue biomarkers. Our pilot data support the potential utility of molecular imaging with FDG and other tracers as an *in vivo* assay to measure pharmacodynamic effects of new anticancer drug therapies and combinations.

## Competing interests

The authors declare that they have no competing interests.

## Authors' contributions

BK participated in the design of the study, performed statistical analyses, and drafted the manuscript. VG, JS, and ER participated in study conduct and recruitment. ES, LP, and XC participated in study coordination, image analysis, and data analysis. HL, DM, and RL conceived of the study; HL and DM participated in the study design and conduct, and drafting of the manuscript. All authors reviewed the manuscript in preparation and approved the final version for submission.
